# Evaluating the Preliminary Efficacy of the TRUST Intervention on Alone-Time and Communication During Well-Adolescent Visits: Quasi-Experimental Study

**DOI:** 10.2196/71433

**Published:** 2025-09-08

**Authors:** Jyu-Lin Chen, Marissa Raymond-Flesch, Thomas J Hoffmann, Chen-Xi Lin, Ling Xu, Kimberly A Koester

**Affiliations:** 1 Department of Family Health Care Nursing School of Nursing University of California, San Francisco San Francisco United States; 2 Division of Adolescent and Young Adult Medicine Department of Pediatrics and the Philip R Lee Institute for Health Policy Studies University of California, San Francisco San Francisco, CA United States; 3 Department of Biostatistics and Epidemiology University of California, San Francisco San Francisco, CA United States; 4 School of Nursing National Defense Medical Center Taipei Taiwan; 5 School of Nursing University of California, San Francisco San Francisco, CA United States; 6 Division of Prevention Science Department of Medicine University of California, San Francisco San Francisco, CA United States

**Keywords:** alone time, technology-based intervention, well-child visits, parent-adolescent communication, sexual and reproductive health, parental monitoring, parental support

## Abstract

**Background:**

Alone time with health care providers is critical for adolescents, and several professional organizations recommend it. Alone time with providers promotes better utilization of health services, empowers adolescents to manage their health, and facilitates discussions on sensitive issues. However, only 40% of adolescents have private conversations with clinicians during visits. The advancement of mobile health technology provides an excellent opportunity to deliver effective interventions to promote adolescent-provider alone time with adolescents, parents, and providers.

**Objective:**

This pilot study aims to explore the preliminary efficacy of a technology-based intervention designed to increase alone time with providers during well-adolescent visits and its impact on trustworthiness, parent-adolescent communication, sexual risk communication, parental monitoring, and parental support before and after the intervention.

**Methods:**

A pre- and postintervention design was used. Participants were recruited through local clinics. After obtaining consent, participants accessed a study website to complete a baseline survey, independently interact with 4 educational modules on a website or cellphone, and complete a post-test survey 1 month after their well-adolescent visits. The surveys assessed alone time with providers, trustworthiness, parent-adolescent communication, sexual risk communication, parental monitoring, and parental support. Mixed model analysis and effect sizes were used to evaluate changes in these outcomes from pre- to postintervention.

**Results:**

Thirty-two dyads (38 adolescents and 32 mothers) participated in this pilot study. About 86% (n=33) of adolescents and 87% (n=28) of mothers completed the pre- and post-test assessments and the intervention. This study found a trend toward an increase in alone time from 81.6% to 84.4%, albeit not statistically significant. Adolescents initiating alone time with providers rose from 6.45% (n=2) to 18.5% (n=5). Over 90% (n=26) of adolescents reported feeling comfortable in one-on-one interactions with providers postintervention. Mixed model analysis revealed significant improvements among adolescents in parental monitoring (*z*=2.93, *P*<.001), sexual risk communication (*z*=3.11, *P*<.001), parent-adolescent communication (*z*=3.11, *P*<.001), open family communication (*z*=2.00, *P*=.04), and parental support (*z*=2.87, *P*<.001). For mothers, significant improvements were found in parental monitoring (*z*=2.45, *P*<.001) and problem family communication (*z*=2.24, *P*=.03).

**Conclusions:**

This pilot study demonstrates promising results regarding the preliminary efficacy of a technology-based intervention to increase alone time with providers during well-adolescent visits and to enhance communication and parenting practices. Improving access to alone time and strengthening communication between parents and adolescents facilitates discussions about sensitive topics, including parental monitoring, sexual and reproductive health, and may strengthen overall satisfaction with health care.

**Trial Registration:**

ClinicalTrials.gov NCT07064070; https://clinicaltrials.gov/study/NCT07064070

## Introduction

### Background

Despite notable improvements in several critical sexual and reproductive health (SRH) protective behaviors in recent years, young people remain considerably vulnerable to sexually transmitted infections (STIs), such as HIV, and unintended pregnancies [1,2]. Individuals aged 15-24 years account for 50% of all newly diagnosed STIs in the United States each year [3]. Adolescents face an elevated risk for STIs due to various factors, such as underutilization of SRH services, inadequate access to sexual health information, medical mistrust, privacy and confidentiality concerns, lack of awareness about their rights to confidential SRH services, and insufficient alone time with health care providers [3-6].

Alone time with health care providers promotes better utilization of health services and empowers adolescents to manage their health, express their emerging autonomy, and discuss sensitive health issues. The American Academy of Pediatrics and the Society for Adolescent Health and Medicine emphasize the importance of providing confidential health services and adolescent-provider alone time during well-adolescent visits [7,8]. Adolescents are more likely to seek health care and discuss sensitive topics when privacy is assured [9,10]. However, a recent medical expenditure panel survey analysis revealed that only 40% of adolescents had alone time with their clinicians during their last visit, and this rate was even lower among adolescents from low-income families, those without insurance, and those of Latinx ethnicity [11]. Another study published in 2022 reported that alone time increases adolescents’ comfort discussing sensitive topics, yet only 31% of adolescents reported experiencing alone time with a provider [12]. A separate study, also published in 2022, showed that some clinics reported rates of adolescent-provider alone as low as 43% [13]. Despite this being a critical mechanism for improving confidential care, few intervention studies have explicitly focused on increasing alone time during well-adolescent visits [14].

Parents play a critical role in adolescents’ health, including promoting alone time with health care providers. Understanding parents’ perspectives on confidential adolescent health care is essential, and it can inform interventions to enhance the uptake of these services. A study found that 58% of parents believe adolescents should have alone time with their clinician [15]. Another study showed that while 89% of parents agreed that adolescents should be able to speak privately with their providers, 61% still preferred to be present in the examination room for the entire visit [6], When parents understand the role of alone time in adolescent health care they may advocate for their adolescent to have access to it, in the same way they might request vaccines or any other evidence-based preventative health service [9,10].

Research suggests that adolescents are accompanied to medical visits by their mothers in the majority of cases; therefore, it is essential to engage them in the intervention. Medical care for adolescents involves a triad of stakeholders, including adolescent health care providers, adolescent patients, and their parents [16]. It is essential to engage parents, especially mothers, in supporting their children’s access to evidence-based health care and encourage alone time. Despite strong recommendations from professional organizations, few interventions focus on increasing alone time to adolescents. Research is needed to examine strategies to effectively increase alone time with health care providers during well-adolescent visits to improve health outcomes generally, as well as SRH outcomes, specifically.

Many parent-adolescent communication interventions focusing on sexual and reproductive health have shown improvements in parent-child communication and parent comfort with communication [17-20], with some resulting in reduced sexual risk behaviors among adolescents [18]. However, most interventions are resource-intensive and lack the feasibility to scale up in the real world. Research indicates that tailored, collaboratively developed communication messages delivered by optimal messengers (eg, peers and trusted adults) through youth-friendly communication channels can significantly encourage protective behaviors (eg, abstinence, consistent and correct condom use, and seeking confidential SRH services) among youth [21,22]. Technology-based interventions provide a promising model for delivering SRH messages and resources that can be scaled and accessed easily and have been proven to promote healthy behavior and can typically be easily accessed [23-25]. A recent web-based program for parents revealed that this type of intervention improved parent-adolescent communication quality and promoted a more positive attitude toward sexual health communication [26]. Another technology-based intervention study for adolescents also found that adolescents reported improvements in intentions for sexual communication, condom use, and self-efficacy to practice safer sex [27]. However, SRH technology-based interventions delivered to adolescents and their parents within the context of primary care visits are limited, and none have focused on increasing alone time with providers.

Parents, especially mothers, can play a crucial role in guiding adolescents toward confidential SRH services and supporting effective communication with health care professionals. Developmentally and culturally tailored interventions that build parent and adolescent self-efficacy related to communicating about SRH, alone time, and parental monitoring are needed. In this study, we developed a technology-based intervention to increase alone time during well-adolescent visits and to promote alone time, communication, and parental monitoring among adolescents and their mothers to address this gap in the literature.

### Theoretical Framework

The social-ecological model (SEM) was used to guide our study. The SEM emphasizes multiple levels of influence on the determinants of health. The levels include individuals (adolescents), interpersonal (mothers), organization (primary care clinics), community (cultural, values, and norms), and organizational (national guidelines) [28,29]. Given that health behaviors both shape and are shaped by the social environment, including interpersonal and organizational levels, the proposed intervention, technology-based resources to increase uptake of sexual health services for teens (TRUST), addressed adolescent’s knowledge, attitude, and skill in navigating sexual health and SRH services, specifically accounting for interpersonal and social influences. The intervention also provided skill-building strategies for adolescents to advocate for alone time with their providers. Similarly, mothers received interactive advice about how to improve communication with adolescents related to SRH and parental monitoring. In addition to the SEM, this study considered the developmental stage of adolescents. Adolescence is a period of rapid physical, social, and cognitive development [30,31]. This dynamic period requires evolving approaches to sexual and reproductive health counseling. Because cognitive and physical development are different based on age, the TRUST intervention was tailored to the development stage of the adolescents enrolled.

### Research Objective

The aim of this pilot study was to evaluate the preliminary efficacy of a technology-based intervention designed to increase adolescents’ alone time with a health care provider during well-adolescent visits. The study also assessed secondary outcomes, including adolescent-mother trust, parent-adolescent communication, sexual risk communication, parental monitoring, and mother support before and after the intervention.

## Methods

### Ethical Considerations

The study was approved by the Institutional Review Board or Ethics Committee of the University of California, San Francisco (20-32013) on November 3, 2021. The clinical trial is registered on ClinicalTrials.gov (NCT07064070).

### Study Design and Setting

The TRUST study used a pre-and post-test design to evaluate the preliminary efficacy of the TRUST intervention in increasing provider and adolescent alone time and improving parent-adolescent communication. All study procedures were carried out in the San Francisco Bay Area and included adolescent and pediatric clinical partners serving low-income and many non-English speaking patient populations, including families of Latinx and Chinese descent.

### Inclusion Criteria

Adolescents could be included in the study if they were aged between 11 and 17 years, able to read and speak English, had access to the Internet (via phone or computer), and had a well-adolescent visit scheduled in the next 6 months. Mothers could be included if they had an adolescent enrolled in this study; spoke and read English, Cantonese, Mandarin, or Spanish; and had access to the Internet.

### Recruitment

Recruitment was conducted at 3 primary care sites in the San Francisco Bay Area. A research champion at each study clinic assisted with recruitment, including by posting flyers in the common areas and exam rooms. Champions identified eligible adolescents with an upcoming well-adolescent visit. The study team sent a formal letter via email inviting the eligible family to enroll in the study. The invitation included (1) a description of the TRUST study objectives (ie, to promote adolescent health outcomes by increasing adolescent use of alone time), (2) a description of the intervention, and (3) details about the duration of the study and data collection, risk and benefits of participation, and safeguards for confidentiality. Interested adolescents and their mothers responded to the study invitation by email, phone, or text, or by accessing a screening form on the TRUST study website. All information was offered in English, Spanish, and Mandarin Chinese. A follow-up phone call or text message was made to interested participants to explain the study in greater detail, answer any questions, and assess eligibility.

### Study Procedures

After obtaining assent from adolescents and consent from mothers, they were directed to the study website, where they could log in to complete the baseline survey assessment. Adolescents and their mothers completed the pretest assessment via a Qualtrics survey between 1 and 3 months before the scheduled well-adolescent visit. After completing the pretest assessment, participants had access to 4 interactive TRUST modules. Adolescents and their mothers were asked to complete these modules before the well-adolescent visit. About 1 month after completing their well-adolescent visit, another Qualtrics survey link was sent to the study participants for post-test assessment.

### Intervention Description

**Figure 1:**
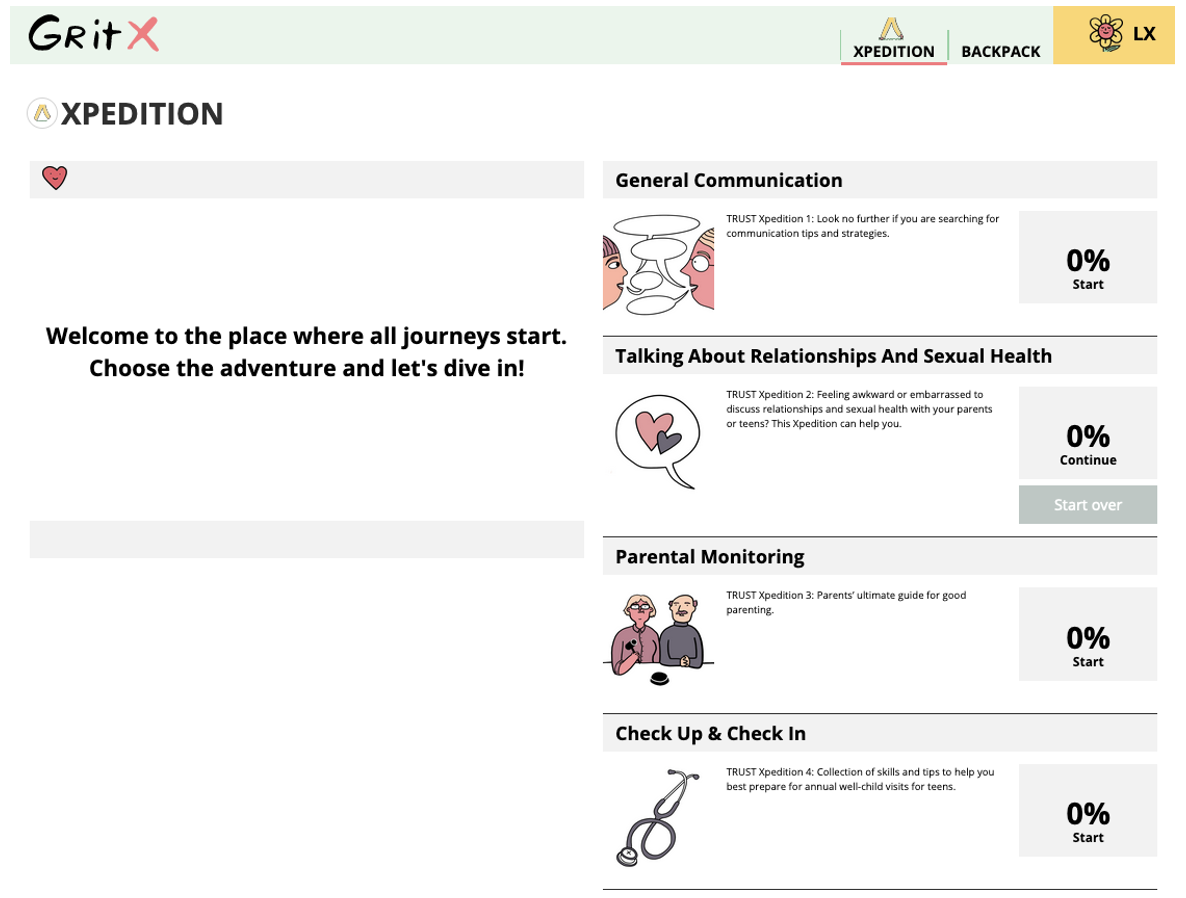
Intervention modules.

The TRUST intervention includes interactive, evidence-based, culturally and developmentally appropriate modules to encourage adolescent alone time with a provider and improve parent-adolescent communication. It was hosted on the GritX website at the University of California, San Francisco [32]. Our modules were developed based on our work with the key stakeholders, including the youth advisory board, parent advisory board, and clinical partner advisory board (including clinicians), and a review of the updated evidence [28]. The modules included: general communication, talking about relationships and sexual health, parental monitoring, and check-up and check-in (content focused on the importance of alone time). Modules were in English, Chinese, or Spanish (Figure 1).

Each interactive module consists of several key features and a roadmap: setting learning goals, assessment of knowledge and confidence, identifying learning topics, review of evidence-based guidelines and tips for the subject, opportunities for practicing skills, and a chance to save favorite information. Each module begins with learning goals and a simple assessment of knowledge on and confidence in the topic. For instance, participants were asked how much they knew about “why alone time between adolescents and health care providers is so important” and how confident they were to engage in “clear and respectful parent-adolescent communication.” There is also a journal section where participants can write notes during their learning, and a virtual “backpack” where participants can store their favorite information. Virtual role-playing was used to engage participants in practicing different communication strategies. In addition, participants earned a badge after completing a module to encourage and motivate continued engagement. Each module took approximately 30-45 minutes to complete. Adolescents and their mothers could review and learn at their own pace and in the language of their choice (English, Chinese, or Spanish) using a smartphone or computer, and they could access resources via a password-protected website. If participants did not access a module within 2 weeks of enrolling in the study, a reminder text was sent to them. A detailed description of the intervention development has been published elsewhere [14].

Each participant received $20 after completing the baseline assessment, $20 after completing the well-adolescent visit, and $30 after completing the post-test assessment.

### Measures

#### Adolescent Self-Reported Alone Time With Providers

To assess adolescents’ alone time with their health care providers during well-adolescent visits, the research team used a specific item from the Young Adult Health Care Survey. The item asked, “Did you get a chance to speak with a doctor or other health provider privately during the last well-adolescent visit?” This question was clarified to mean one-on-one communication without parents or other individuals present in the room. The Young Adult Health Care Survey is a well-established survey that has demonstrated reliability and validity [33]. Participants also responded to who initiated the alone time with the primary care provider.

#### Measures of Trustworthiness

The construct of perceived trustworthiness was assessed using 3 items for adolescents and their mothers. The three items were as follows: (1) I can trust my mother/adolescent when we talk, (2) my mother/adolescent keeps her promises to me, and (3) my mother/adolescent is honest with me. Each item was rated on a 5-point Likert scale ranging from 1 (strongly agree) to 5 (strongly disagree). This scale allowed participants to express varying degrees of agreement or disagreement with each statement. The reliability and validity of these items are supported by previous research [34].

#### Parent-Adolescent Communication Scale

The parent-adolescent communication scale, developed by Olson (1985) [35], consists of 20 items that measure the quality of communication between adolescents and their parents. It is designed for both parents and adolescents to complete. Each item was rated on a 5-point Likert scale ranging from “strongly disagree” to “strongly agree,” generating a total score and 2 subscale scores. The open family communication subscale reflects feelings of free expression and understanding in parent-adolescent interactions (eg, “When I ask questions, I get honest answers from my mother/father”). The problems in family communication subscale measures negative interaction patterns and hesitancy to disclose concerns (eg, “My mother/father tends to say things to me that would be better left unsaid”). Higher scores indicate better parent-adolescent communication. The parent-adolescent communication scale demonstrates good internal consistency [36,37].

#### Parent-Adolescent Sexual Risk Communication Scale III

The parent-adolescent sexual risk communication scale III, developed by Hutchinson [38], is an 8-item scale used to assess the extent of sexual risk communication between adolescents and their parents. The scale includes separate scales for adolescents to report communication with their mothers and fathers. In this study, we asked for communication with the mother only. Adolescents were asked to report how much information their mother communicated with them on a 5-point scale (1=none; 2=a little; 3=some; 4=a lot; and 5=extensive) and on the following topics between the ages of 10-18 years: birth control, sexually transmitted diseases (STDs), HIV/AIDS, condoms, how to protect yourself from HIV/AIDS, postponing or not having sex, peer pressure to have sex, and how to handle sexual pressure. The total scores range from 8 to 40, with higher scores indicating more extensive communication. Appropriate reliability and validity have been reported [38].

#### Parental Monitoring

This validated scale assesses the degree to which parents are informed about various aspects of their child’s life [39]. This construct was measured using 9 questions that parents and adolescents answered on a 5-point Likert scale, ranging from 1 (no, never) to 5 (yes, almost always). Sample questions include, “Do your parents: know what you do during your free time? Know who you have as friends during your free time? Usually, know what type of homework you have? Know what you spend your money on?”

#### Parental Support (Mothers Only)

Mothers reported how often they support their child using a 3-item 4-point Likert scale (never to always). Sample items include, “How often do you let your child know you care about them?” and “How often do you listen to your child carefully?” Higher values indicate more supportive parenting. Adequate reliability has been reported [26].

### Statistical Analysis

This pilot study aimed to explore the preliminary efficacy of the intervention. Descriptive statistics include the mean and proportion of participant demographic data and outcome variables. A mixed model analysis with intention to treat estimated the intervention’s preliminary efficacy, controlling for age. This method allows us to use all available data, regardless of missing data. Six families with 2 adolescents were included in this study; family unit identification and individual unit identification were included as random effect parameters in the mixed model analyses. Effect sizes for pre- and postintervention outcome variables were assessed using the Cohen *d* index. Effect sizes were categorized as small (<0.20), medium (0.50), and large (>0.80). Statistical significance was determined with a *P* value set at <.05. All analyses were performed using Stata version 18.0.

## Results

### Sample Characteristics

Thirty-two dyads (38 adolescents and 32 mothers) participated in this study. The average age was 14.5 (SD 1.81) years and 49.06 (SD 7.9) years for adolescents and their mothers, respectively. Among the mothers, 10 out of 32 (31%) reported having a master’s degree or higher, and 13 out of 32 (41%) had an annual household income greater than $120,001 (Table 1). Approximately 12 families identified themselves as European White (40%), 7 as Latinx (21.8%), and 13 as Asian (40.5%). Additionally, about 86% (n=33) of adolescents and 87% (n=28) of mothers completed the pre- and post-test assessments and the intervention (Table 2).

**Table 1:** Participant demographics.

Variable		Value
**Age (years), mean (SD)**		
	Adolescents (n=38)	14.53 (1.81)
	Mothers (n=32)	49.06 (7.83)
**Occupation (mother), n (%)**		
	Administrative and office roles	7 (23)
	Customer service	4 (13)
	Education and training	4 (13)
	Media and creative	2 (6)
	Health care and therapy	3 (10)
	Science and research	1 (3)
	Business	5 (16)
	Library clerk	1 (3)
	Retired hairstylist	1 (3)
	School crossing guard	1 (3)
	Student	1 (3)
	School bus driver	1 (3)
**Annual household income, n (%)**		
	<$20,000	2 (6)
	$20,000 to $80,000	10 (31)
	$80,001 to $120,000	6 (19)
	>$120,001	14 (44)
**Education (mother), n (%)**		
	12th grade or below	6 (19)
	Some college or vocational degree	3 (9)
	Associate degree	3 (9)
	Bachelor’s degree	9 (28)
	Master’s degree or above	10 (31)
	Refused	1 (3)

**Table 2:** Alone time reported by adolescents.

Variable		Baseline, n (%)	Follow-up, n (%)	Chi-square (*df)*		*P* value	
**Adolescent talks with a provider privately**					0.10 (3)		.76
	Yes	31 (82)	27 (84)				
	No	7 (18)	5 (16)				
**Initiated the alone time (adolescents)**					2.02 (5)		.36
	Provider	28 (90)	21 (78)				
	Adolescent	2 (6)	5 (19)				
	Mother	1 (3)	1 (4)				
**How comfortable was it for you to be one-on-one with your provider**					N/A^a^		N/A
	Very comfortable	N/A	10 (36)				
	Somewhat comfortable	N/A	16 (57)				
	Somewhat uncomfortable	N/A	1 (4)				
	Very uncomfortable	N/A	1 (4)				

^a^Not applicable.

### Adolescent-Reported Alone Time and Comfort Level

In terms of alone time with providers, 27 out of 32 adolescents (84%) reported having alone time with the provider at their well-adolescent visit after the intervention, compared to 31 out of 38 (82%) at baseline (χ^2^_3_=.01, *P*=.76). The number of adolescents who initiated alone time with a provider increased from 2 out of 31 (6%) at baseline to 5 out of 27 (19%) at follow-up. The number of mothers who initiated alone time remained nearly the same: 1 out of 31 (3%) at baseline and 1 out of 27 (4%) at follow-up. Regarding adolescents’ comfort levels in one-on-one interaction with the provider at follow-up, 16 out of 28 (57%) felt somewhat comfortable, and 10 out of 28 adolescents (36%) reported feeling very comfortable (Table 2).

### Communication Outcome, Parental Monitoring, and Parental Support

Mixed model analysis controlling for age revealed significant improvements among adolescents in sexual risk communication (*z*=3.11, *P*<.001), parent-adolescent communication (*z*=3.11, *P*<.001), open family communication (*z*=2.00; *P*=.04), parental monitoring (*z*= 2.93, *P*<.001), and parental support (*z*=2.87, *P*<.001; Table 3). For mothers, significant improvements were found in parent-adolescent communication: problem family communication (*z*=2.24, *P*=.03) and parental monitoring (*z*=2.45, *P*<.001; Table 4).

**Table 3:** Mixed effects models results for adolescents.

Outcome			Coefficient (β)		Standard error		*z* score		*P* value		95% CI
**Trustworthiness**											
	Time	0.61		0.39		1.54		.12		–0.16 to 1.37	
	Age	–0.17		0.17		–0.99		.32		–0.51 to 0.17	
**Parental monitoring**											
	Time	0.29		0.10		2.93		<.001		0.10 to 0.49	
	Age	–0.08		0.04		–2.10		.04		–0.16 to –0.01	
**Sexual risk communication**											
	Time	2.98		0.96		3.11		<.001		1.10 to 4.85	
	Age	0.76		0.69		1.09		.27		–0.60 to 2.12	
**Parent-adolescent communication**											
	Time	4.55		1.46		3.11		<.001		1.69 to 7.41	
	Age	–1.14		0.98		–1.16		.25		–3.06 to 0.78	
**Open family communication subscale**											
	Time	1.88		0.94		2.00		.04		0.04 to 3.73	
	Age	–0.80		0.58		–1.38		.17		–1.93 to 0.34	
**Problem family communication subscale**											
	Time	–0.41		0.94		–0.44		.66		–2.25 to 1.42	
	Age	–0.44		0.52		–0.85		.40		–1.47 to 0.58	
**Parental support**											
	Time	0.63		0.22		2.87		<.001		0.20 to 1.07	
	Age	–0.28		0.12		–2.28		.02		–0.52 to –0.04	

**Table 4:** Mixed effects model results for mothers.

Outcome		Coefficient (β)	Standard error	*z* score	*P* value	95% CI
**Trustworthiness**						
	Time	0.48	0.54	0.90	.37	–0.57 to 1.53
	Age	–0.06	0.05	–1.28	.20	–0.15 to 0.03
**Parental monitoring**						
	Time	0.46	0.13	3.45	<.001	0.20 to 0.72
	Age	–0.01	0	–1.32	.19	–0.03 to 0.01
**Parent-adolescent communication**						
	Time	2.80	2.00	1.40	.16	–1.12 to 6.72
	Age	–0.23	0.22	–1.05	.30	–0.66 to 0.20
**Open family communication subscale**						
	Time	–0.06	1.20	–0.05	>.99	–2.42 to 2.29
	Age	–0.15	0.12	–1.24	.22	–0.37 to 0.08
**Problem family communication subscale**						
	Time	2.64	1.18	2.24	.03	0.33 to 4.95
	Age	–0.08	0.13	–0.66	.51	–0.34 to 0.17
**Parental support**						
	Time	0.17	0.33	0.52	.60	–0.47 to 0.81
	Age	–0.05	0.03	–1.85	.06	–0.10 to 0.00

### Effect Size Analysis

A small effect was found for the alone time variable (Cramér V=0.05). The results indicated varying effect sizes on secondary outcomes based on the Cohen *d* index for pre- and post-tests. For adolescents, small effect sizes were found in trustworthiness, sexual risk communication, problem in the family communication subscale, parent-adolescent communication, and parental support total score. A medium effect size was observed in parental monitoring. For mothers, small effect sizes were found in several domains, including problem-free family communication, parent-adolescent communication total score, and parental support total score. A large effect size (Cohen *d*=1.05) was observed in parental monitoring (Table 5).

**Table 5:** Outcome data between baseline and follow-up.

Variable		Baseline (n=70)			Follow-up (n=59)			Effect size (Cohen *d*)^a^
		n	Mean (SD)	n		Mean (SD)		
**Trustworthiness**								
	Adolescents	38	12.10 (2.31)	30		12.80 (2.10)	0.31	
	Mothers	31	12.87 (2.91)	27		13.33 (2.13)	0.18	
**Parental monitoring**								
	Adolescents	38	3.67 (0.57)	31		3.94 (0.51)	0.50	
	Mothers^b^	32	3.79 (0.48)	27		4.29 (0.47)	1.05	
**Sexual risk communication^a^**								
	Adolescents	37	17.86 (8.07)	31		20.48 (8.31)	0.32	
**Parent-Adolescent Communication**								
	Adolescents^b^	37	36.51 (7.83)	31		37.52 (6.04)	0.14	
	Mothers	31	37.81 (5.59)	27		38.26 (5.41)	0.08	
**Problem with family communication**								
	Adolescents	37	31.84 (6.64)	31		30.61 (5.72)	0.20	
	Mothers^b^	31	34.29 (5.42)	27		36.56 (6.14)	0.39	
**Open family communication**								
	Adolescents^b^	37	65.32 (12.43)	31		68.12 (10.61)	0.24	
	Mothers	31	72.10 (9.78)	27		74.81 (10.44)	0.27	
**Parental support**								
	Adolescents^b^	38	9.87 (1.66)	31		10.22 (1.48)	0.22	
	Mothers	32	10.28 (1.14)	27		10.63 (1.33)	0.28	

^a^Small effect: Cohen *d*=0.2-0.49; medium effect: Cohen *d*=0.5-79; large effect: Cohen *d*=0.8-1.29; very large effect: Cohen *d*≥1.3.

^b^Significant at mixed model analysis.

## Discussion

### Principal Findings

This pilot study examined the preliminary efficacy of a technology-based intervention on alone time with a provider during well-adolescent visits by assessing trustworthiness, parent-adolescent communication, sexual risk communication, parental monitoring, and parental support before and after the intervention. We found an increase in reports of alone time with the provider at the last well-adolescent visit, increasing from 81.6% to 84.4%, although there was no statistical significance. We also found an improvement in the percentage of adolescents initiating alone time with a provider, which increased from 6.45% to 18.5%. Importantly, over 90% of adolescents reported feeling comfortable or very comfortable in one-on-one interactions with the provider after the intervention. In addition, this study revealed significant improvements in communication outcomes, parental monitoring, and parental support among adolescents. Small effect sizes were also noted in trustworthiness among adolescents. Significant improvements were also observed in parental monitoring and problem family communication among mothers. These findings suggest there is promise in using the technology-based TRUST intervention to improve adolescents’ alone time at well-adolescent visits and improve parent-adolescent communication and parenting practices.

Empowering adolescents to have private conversations with health care providers is important because alone time during well-adolescent visits has been shown to facilitate discussions on sensitive health topics, including SRH, and ensure confidentiality in these matters [9,10,40]. Additionally, alone time with providers can enhance adolescents’ satisfaction with their health care, which is crucial for ensuring continuity of care and better access to necessary health services [41]. Our pilot showed a particularly high level of reported alone time with primary care providers during well-adolescent visits at baseline, significantly surpassing the figures reported by Grilo et al [42], which ranged from 44% to 55% among nearly 2000 US youths. This suggests that our collaborating clinical sites adhere to professional recommendations to offer alone time. Despite this high baseline, we observed a 3% increase in alone time and a remarkable 12% increase in adolescents initiating alone time with their provider, suggesting the positive impact of the intervention. Although our intervention targeted adolescents and mothers, it may have indirectly influenced provider behavior by encouraging adolescent self-advocacy or maternal prompting. In addition, while clinicians were not directly involved in accessing the intervention, we engaged them as an advisory group during its design phase to strengthen its impact. However, the factors driving provider-initiated alone time are complex and likely rooted in structural factors, clinic norms, and providers’ own perspectives. Future studies should consider multilevel interventions that directly engage providers to enhance adolescent alone time during visits.

Our results reveal a low frequency of mother-initiated alone time, suggesting potential attitudinal or cultural influences. This underscores the importance of promoting the advocacy role of adolescents in requesting alone time. Future research should incorporate stronger parental components to better educate parents about the importance of encouraging adolescent-private time with health care providers.

In addition to the increase in alone time and the initiation of alone time by the adolescent, we also found improvements in various communication domains (parent and adolescent communication and SRH communication) and parenting areas (monitoring and support) reported by the adolescent after the intervention. Mothers reported an increase in parental monitoring and an increase in communicating about family problems. However, most observed effect sizes were small to medium. One possible explanation is the short interval between the intervention and follow-up, which may not allow sufficient time for meaningful changes to emerge in trust-building or established family communication patterns. Another possibility is that the brief, nonreinforced nature of the intervention may have limited its impact.

Strengthening communication skills between mothers and adolescents is particularly important. Improved parent-adolescent communication may enhance adolescents’ ability to engage in appropriate SRH behaviors, use confidential health services when needed, and communicate more effectively with health care providers [15,19]. These findings are consistent with prior studies, suggesting that web-based programs for parents can improve the quality of parent-adolescent communication and foster a more positive attitude toward sexual health communication [26,27]. These results are consistent with 3 studies reporting an improvement in SRH communication after receiving the technology-based intervention [26,43,44], yet 2 studies found no significant difference [26,45].

### Limitations

This pilot study reports the preliminary efficacy of a technology-based intervention, yet these results must be interpreted cautiously, as several limitations should be noted. First, the pilot study included a small sample size, comprising 32 dyads (38 adolescents and 32 mothers). This limited sample size may restrict the generalizability of the results to broader populations. Our study participants comprised mothers with high educational attainment, and a large portion reported high annual household income (41% earning over $120,001, the median household income in the San Francisco Area). This study sample may only reflect the San Francisco Bay Area population.

Second, our study used a quasi-experimental design without a control group; therefore, the results may be partially attributed to the intervention. The use of self-reported data may also increase potential bias. It is recommended that future studies include larger, more diverse samples and control groups, direct observation of alone time, and an extended follow-up period so that a comprehensive understanding of the intervention’s efficacy can be established.

Third, while the TRUST intervention aimed to promote adolescent-provider alone time, it did not explicitly address psychological drivers of mistrust. The adolescent-mother trust, our secondary outcome, also did not show statistically significant improvement. Future studies should incorporate trust-building components to align more closely with the TRUST acronym.

Moreover, our baseline assessment of adolescent-provider interactions relied on retrospective self-reporting about participants’ last health care encounter. This approach introduces methodological limitations such as potential recall bias due to varying time lapses between the actual encounter and survey completion. Future studies should implement real-time data collection after the actual encounter.

Finally, our study’s high baseline alone time rate may reflect strong pre-existing clinic practices, as we mentioned earlier, which could have limited our ability to detect statistically significant changes, indicating a potential ceiling effect. Future studies should adopt a more diverse recruitment strategy to capture a wider range of baseline adolescents’ behaviors.

Based on the literature and our clinical and research experiences, mothers are usually the primary caregivers of their adolescents. Therefore, this study included both adolescents and their mothers, forming a mother-adolescent dyad. A preliminary analysis indicated strong parental involvement from the mothers. However, due to the small sample size, it will be challenging to assess the impact of parental involvement on the outcomes. Future studies with larger sample sizes should include mediation analysis to evaluate the effect of parental involvement on outcomes. Despite these limitations, our technology-based, interactive intervention with tailored content actively equipped adolescents to advocate for alone time and improved adolescent- mother communication.

### Conclusions

Our culturally grounded and technology-based intervention is one of the first aimed at improving rates of and comfort with provider-adolescent patient alone time. This pilot study demonstrates promising results regarding increased alone time with providers during well-adolescent visits and enhanced communication and parenting practices. These findings align with existing research on technology-based interventions and suggest that such approaches can effectively promote alone time with providers, strengthen communication, and support adolescents in engaging in private consultations, a foundational step toward promoting lifelong engagement in preventative health care.

## Abbreviations

SEM: social-ecological model
SRH: sexual and reproductive health
STI: sexually transmitted infection
TRUST: technology-based resources to increase uptake of sexual health services for teens
